# Ethics review of big data research: What should stay and what should be reformed?

**DOI:** 10.1186/s12910-021-00616-4

**Published:** 2021-04-30

**Authors:** Agata Ferretti, Marcello Ienca, Mark Sheehan, Alessandro Blasimme, Edward S. Dove, Bobbie Farsides, Phoebe Friesen, Jeff Kahn, Walter Karlen, Peter Kleist, S. Matthew Liao, Camille Nebeker, Gabrielle Samuel, Mahsa Shabani, Minerva Rivas Velarde, Effy Vayena

**Affiliations:** 1 Health Ethics and Policy Lab, Department of Health Sciences and Technology, ETH Zürich, Hottingerstrasse 10 (HOA), 8092 Zürich, Switzerland; 2 The Ethox Centre, Department of Population Health, University of Oxford, Oxford, UK; 3 School of Law, University of Edinburgh, Edinburgh, UK; 4 Brighton and Sussex Medical School, Brighton, UK; 5 Biomedical Ethics Unit, Department of Social Studies of Medicine, McGill University, Montreal, Canada; 6 Johns Hopkins Berman Institute of Bioethics, Baltimore, USA; 7 Mobile Health Systems Lab, Department of Health Sciences and Technology, ETH Zürich, Zürich, Switzerland; 8 Cantonal Ethics Committee Zürich, Zürich, Switzerland; 9 Center for Bioethics, Department of Philosophy, New York University, New York, USA; 10 Research Center for Optimal Digital Ethics in Health (ReCODE Health), Herbert Wertheim School of Public Health and Longevity Science, University of California, San Diego, USA; 11 Department of Global Health and Social Medicine, King’s College London, London, UK; 12 Faculty of Law and Criminology, Ghent University, Ghent, Belgium; 13 Department of Radiology and Medical Informatics, Faculty of Medicine, University of Geneva, Geneva, Switzerland

**Keywords:** Big data, Research ethics, Ethics, IRBs, RECs, Ethics review, Biomedical research

## Abstract

**Background:**

Ethics review is the process of assessing the ethics of research involving humans. The Ethics Review Committee (ERC) is the key oversight mechanism designated to ensure ethics review. Whether or not this governance mechanism is still fit for purpose in the data-driven research context remains a debated issue among research ethics experts.

**Main text:**

In this article, we seek to address this issue in a twofold manner. First, we review the strengths and weaknesses of ERCs in ensuring ethical oversight. Second, we map these strengths and weaknesses onto specific challenges raised by big data research. We distinguish two categories of potential weakness. The first category concerns persistent weaknesses, i.e., those which are not specific to big data research, but may be exacerbated by it. The second category concerns novel weaknesses, i.e., those which are created by and inherent to big data projects. Within this second category, we further distinguish between purview weaknesses related to the ERC’s scope (e.g., how big data projects may evade ERC review) and functional weaknesses, related to the ERC’s way of operating. Based on this analysis, we propose reforms aimed at improving the oversight capacity of ERCs in the era of big data science.

**Conclusions:**

We believe the oversight mechanism could benefit from these reforms because they will help to overcome data-intensive research challenges and consequently benefit research at large.

## Background

The debate about the adequacy of the Ethics Review Committee (ERC) as the chief oversight body for big data studies is partly rooted in the historical evolution of the ERC. Particularly relevant is the ERC’s changing response to new methods and technologies in scientific research. ERCs—also known as Institutional Review Boards (IRBs) or Research Ethics Committees (RECs)—came to existence in the 1950s and 1960s [1]. Their original mission was to protect the interests of human research participants, particularly through an assessment of potential harms to them (e.g., physical pain or psychological distress) and benefits that might accrue from the proposed research. ERCs expanded in scope during the 1970s, from participant protection towards ensuring valuable and ethical human subject research (e.g., having researchers implement an informed consent process), as well as supporting researchers in exploring their queries [2].

Fast forward fifty years, and a lot has changed. Today, biomedical projects leverage unconventional data sources (e.g., social media), partially inscrutable data analytics tools (e.g., machine learning), and unprecedented volumes of data [3–5]. Moreover, the evolution of research practices and new methodologies such as post-hoc data mining have blurred the concept of ‘*human subject’* and elicited a shift towards the concept of *data subject*—as attested in data protection regulations. [6, 7]. With data protection and privacy concerns being in the spotlight of big data research review, language from data protection laws has worked its way into the vocabulary of research ethics. This terminological shift further reveals that big data, together with modern analytic methods used to interpret the data, creates novel dynamics between researchers and participants [8]. Research data repositories about individuals and aggregates of individuals are considerably expanding in size. Researchers can remotely access and use large volumes of potentially sensitive data without communicating or actively engaging with study participants. Consequently, participants become more vulnerable and subjected to the research itself [9]. As such, the *nature of risk* involved in this new form of research changes too. In particular, it moves from the risk of physical or psychological harm towards the risk of informational harm, such as *privacy breaches* or *algorithmic discrimination* [10]. This is the case, for instance, with projects using data collected through web search engines, mobile and smart devices, entertainment websites, and social media platforms. The fact that health-related research is leaving hospital labs and spreading into online space creates novel opportunities for research, but also raises novel challenges for ERCs. For this reason, it is important to re-examine the fit between new data-driven forms of research and existing oversight mechanisms [11].

The suitability of ERCs in the context of big data research is not merely a theoretical puzzle but also a practical concern resulting from recent developments in data science. In 2014, for example, the so-called ‘emotional contagion study’ received severe criticism for avoiding ethical oversight by an ERC, failing to obtain research consent, violating privacy, inflicting emotional harm, discriminating against data subjects, and placing vulnerable participants (e.g., children and adolescents) at risk [12, 13]. In both public and expert opinion [14], a responsible ERC would have rejected this study because it contravened the research ethics principles of preventing harm (in this case, emotional distress) and adequately informing data subjects. However, the protocol adopted by the researchers was not required to undergo ethics review under US law [15] for two reasons. First, the data analyzed were considered non-identifiable, and researchers did not engage directly with subjects, exempting the study from ethics review. Second, the study team included both scientists affiliated with a public university (Cornell) and Facebook employees. The affiliation of the researchers is relevant because—in the US and some other countries—privately funded studies are not subject to the same research protections and ethical regulations as publicly funded research [16]. An additional example is the 2015 case in which the United Kingdom (UK) National Health Service (NHS) shared 1.6 million pieces of identifiable and sensitive data with Google DeepMind. This data transfer from the public to the private party took place legally, without the need for patient consent or ethics review oversight [17]. These cases demonstrate how researchers can pursue potentially risky big data studies without falling under the ERC’s purview. The limitations of the regulatory framework for research oversight are evident, in both private and public contexts.

The gaps in the ERC’s regulatory process, together with the increased sophistication of research contexts—which now include a variety of actors such as universities, corporations, funding agencies, public institutes, and citizens associations—has led to an increase in the range of oversight bodies. For instance, besides traditional university ethics committees and national oversight committees, funding agencies and national research initiatives have increasingly created internal ethics review boards [18, 19]. New participatory models of governance have emerged, largely due to an increase in subjects’ requests to control their own data [20]. Corporations are creating research ethics committees as well, modelled after the institutional ERC [21]. In May 2020, for example, Facebook welcomed the first members of its Oversight Board, whose aim is to review the company’s decisions about content moderation [22]. Whether this increase in oversight models is motivated by the urge to fill the existing regulatory gaps, or whether it is just ‘ethics washing’, is still an open question. However, other types of specialized committees have already found their place alongside ERCs, when research involves international collaboration and data sharing [23]. Among others, data safety monitoring boards, data access committees, and responsible research and innovation panels serve the purpose of covering research areas left largely unregulated by current oversight [24].

The data-driven digital transformation challenges the purview and efficacy of ERCs. It also raises fundamental questions concerning the role and scope of ERCs as the oversight body for ethical and methodological soundness in scientific research.^1^ Among these questions, this article will explore whether ERCs are still capable of their intended purpose, given the range of novel (maybe not categorically new, but at least different in practice) issues that have emerged in this type of research. To answer this question, we explore some of the challenges that the ERC oversight approach faces in the context of big data research and review the main strengths and weaknesses of this oversight mechanism. Based on this analysis, we will outline possible solutions to address current weaknesses and improve ethics review in the era of big data science.

## Main text

### Strengths of the ethics review via ERC

Historically, ERCs have enabled cross disciplinary exchange and assessment [27]. ERC members typically come from different backgrounds and bring their perspectives to the debate; when multi-disciplinarity is achieved, the mixture of expertise provides the conditions for a solid assessment of advantages and risks associated with new research. Committees which include members from a variety of backgrounds are also suited to promote projects from a range of fields, and research that cuts across disciplines [28]. Within these committees, the reviewers’ expertise can be paired with a specific type of content to be reviewed. This one-to-one match can bring timely and, ideally, useful feedback [29]. In many countries (e.g., European countries, the United States (US), Canada, Australia), ERCs are explicitly mandated by law to review many forms of research involving human participants; moreover, these laws also describe how such a body should be structured and the purview of its review [30, 31]. In principle, ERCs also aim to be representative of society and the research enterprise, including members of the public and minorities, as well as researchers and experts [32]. And in performing a gatekeeping function to the research enterprise, ERCs play an important role: they recognize that both experts and lay people should have a say, with different views to contribute [33].

Furthermore, the ERC model strives to ensure independent assessment. The fact that ERCs assess projects “from the outside” and maintain a certain degree of objectivity towards what they are reviewing, reduces the risk of overlooking research issues and decreases the risk for conflicts of interest. Moreover, being institutionally distinct—for example, being established by an organization that is distinct from the researcher or the research sponsor—brings added value to the research itself as this lessens the risk for conflict of interest. Conflict of interest is a serious issue in research ethics because it can compromise the judgment of reviewers. Institutionalized review committees might particularly suffer from political interference. This is the case, for example, for universities and health care systems (like the NHS), which tend to engage “in house” experts as ethics boards members. However, ERCs that can prove themselves independent are considered more trustworthy by the general public and data subjects; it is reassuring to know that an independent committee is overseeing research projects [34].

The ex-ante (or pre-emptive) ethical evaluation of research studies is by many considered the standard procedural approach of ERCs [35]. Though the literature is divided on the usefulness and added value provided by this form of review [36, 37], ex-ante review is commonly used as a mechanism to ensure the ethical validity of a study design before the research is conducted [38, 39]. Early research scrutiny aims at risk-mitigation: the ERC evaluates potential research risks and benefits, in order to protect participants’ physical and psychological well-being, dignity, and data privacy. This practice saves researchers’ resources and valuable time by preventing the pursuit of unethical or illegal paths [40]. Finally, the ex-ante ethical assessment gives researchers an opportunity to receive feedback from ERCs, whose competence and experience may improve the research quality and increase public trust in the research [41].

All *strengths* mentioned in this section are strengths of the ERC model in principle. In practice, there are many ERCs that are not appropriately interdisciplinary or representative of the population and minorities, that lack independence from the research being reviewed, and that fail to improve research quality, and may in fact hinder it. We now turn to consider some of these weaknesses in more detail.

### Weaknesses of the ethics review via ERC

**Fig. 1:**
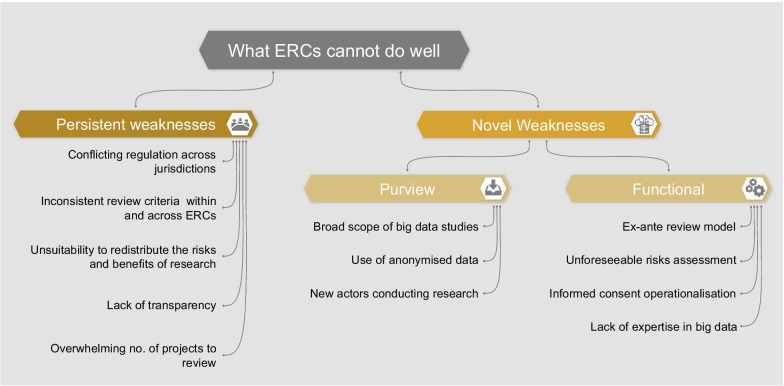
Weaknesses of the ERCs

In order to assess whether ERCs are adequately equipped to oversee big data research, we must consider the weaknesses of this model. We identify two categories of weaknesses which are described in the following section and summarized in Fig. 1:

- *Persistent weaknesses*: those existing in the current oversight system, which could be exacerbated by big data research
- *Novel weaknesses*: those brought about by and specific to the nature of big data projects
  Within this second category of novel weaknesses, we further differentiate between:
- *Purview weaknesses*: reasons why some big data projects may bypass the ERCs’ purview
- *Functional weaknesses*: reasons why some ERCs may be inadequate to assess big data projects specifically

We base the conceptual distinction between persistent and novel weaknesses on the fact that big data research diverges from traditional biomedical research in many respects. As previously mentioned, big data projects are often broad in scope, involve new actors, use unprecedented methodologies to analyze data, and require specific expertise. Furthermore, the peculiarities of big data itself (e.g., being large in volume and from a variety of sources) make data-driven research different in practice from traditional research. However, we should not consider the category of “novel weaknesses” a closed category. We do not argue that weaknesses mentioned here do not, at least partially, overlap with others which already exist. In fact, in almost all cases of ‘novelty’, (i) there is some link back to a concept from traditional research ethics, and (ii) some thought has been given to the issue outside of a big data or biomedical context (e.g., the problem of ERCs’ expertise has arisen in other fields [42]). We believe that by creating conceptual clarity about novel oversight challenges presented by big data research, we can begin to identify tailored reforms.

### Persistent weaknesses

As regulation for research oversight varies between countries, ERCs often suffer from a lack of harmonization. This weakness in the current oversight mechanism is compounded by big data research, which often relies on multi-center international consortia. These consortia in turn depend on approval by multiple oversight bodies demanding different types of scrutiny [43]. Furthermore, big data research may give rise to collaborations between public bodies, universities, corporations, foundations, and citizen science cooperatives. In this network, each stakeholder has different priorities and depends upon its own rules for regulation of the research process [44–46]. Indeed, this expansion of regulatory bodies and aims does not come with a coordinated effort towards agreed-upon review protocols [47]. The lack of harmonization is perpetuated by academic journals and funding bodies with diverging views on the ethics of big data. If the review bodies which constitute the “ethics ecosystem” [19] do not agree to the same ethics review requirements, a big data project deemed acceptable by an ERC in one country may be rejected by another ERC, within or beyond the national borders.

In addition, there is inconsistency in the assessment criteria used within and across committees. Researchers report subjective bias in the evaluation methodology of ERCs, as well as variations in ERC judgements which are not based on morally relevant contextual considerations [48, 49]. Some authors have argued that the probability of research acceptance among experts increases if some research peer or same-field expert sits on the evaluation committee [50, 51]. The judgement of an ERC can also be influenced by the boundaries of the scientific knowledge of its members. These boundaries can impact the ERC’s approach towards risk taking in unexplored fields of research [52]. Big data research might worsen this problem since the field is relatively new, with no standardized metric to assess risk within and across countries [53]. The committees do not necessarily communicate with each other to clarify their specific role in the review process, or try to streamline their approach to the assessment. This results in unclear oversight mandates and inconsistent ethical evaluations [27, 54].

Additionally, ERCs may fall short in their efforts to justly redistribute the risks and benefits of research. The current review system is still primarily tilted toward protecting the interests of individual research participants. ERCs do not consistently assess societal benefit, or risks and benefits in light of the overall conduct of research (balancing risks for the individual with collective benefits). Although demands on ERCs vary from country to country [55], the ERC approach is still generally tailored towards traditional forms of biomedical research, such as clinical trials and longitudinal cohort studies with hospital patients. These studies are usually narrow in scope and carry specific risks only for the participants involved. In contrast, big data projects can impact society more broadly. As an example, computational technologies have shown potential to determine individuals’ sexual orientation by screening facial images [56]. An inadequate assessment of the common good resulting from this type of study can be socially detrimental [57]. In this sense, big data projects resemble public health research studies, with an ethical focus on the common good over individual autonomy [58]. Within this context, ERCs have an even greater responsibility to ensure the just distribution of research benefits across the population. Accurately determining the social value of big data research is challenging, as negative consequences may be difficult to detect before research begins. Nevertheless, this task remains a crucial objective of research oversight.

The literature reports examples of the failure of ERCs to be accountable and transparent [59]. This might be the result of an already unclear role of ERCs. Indeed, the ERCs practices are an outcome of different levels of legal, ethical, and professional regulations, which largely vary across jurisdictions. Therefore, some ERCs might function as peer counselors, others as independent advisors, and still others as legal controllers. What seems to be common across countries, though, is that ERCs rarely disclose their procedures, policies, and decision-making process. The ERCs’ “secrecy” can result in an absence of trust in the ethical oversight model [60].This is problematic because ERCs rely on public acceptance as accountable and trustworthy entities [61]. In big data research, as the number of data subjects is exponentially greater, a lack of accountability and an opaque deliberative process on the part of ERCs might bring even more significant public backlash. Ensuring truthfulness of the stated benefits and risks of research is a major determinant of trust in both science and research oversight. Researchers are another category of stakeholders negatively impacted by poor communication and publicity on the part of the ERC. Commentators have shown that ERCs often do not clearly provide guidance about the ethical standards applied in the research review [62]. For instance, if researchers provide unrealistic expectations of privacy and security to data subjects, ERCs have an institutional responsibility to flag those promises (e.g., about data security and the secondary-uses of subject data), especially when the research involves personal and high sensitivity data [63]. For their part, however, ERCs should make their expectations and decision-making processes clear.

Finally, ERCs face the increasing issue of being overwhelmed by the number of studies to review [64, 65]. Whereas ERCs originally reviewed only human subjects research happening in natural sciences and medicine, over time they also became the ethical body of reference for those conducting human research in the social sciences (e.g., in behavioral psychology, educational sciences, etc.). This increase in demand creates pressure on ERC members, who often review research *pro bono* and on a voluntary basis. The wide range of big data research could exacerbate this existing issue. Having more research to assess and less time to accomplish the task may negatively impact the quality of the ERC’s output, as well as increase the time needed for review [66]. Consequently, researchers might carry out potentially risky studies because the relevant ethical issues of those studies were overlooked. Furthermore, research itself could be significantly delayed, until it loses its timely scientific value.

### Novel weaknesses: purview weaknesses

To determine whether the ERC is still the most fit-for-purpose entity to oversee big data research, it is important to establish under which conditions big data projects fall under the purview of ERCs.

Historically, research oversight has primarily focused on human subject research in the biomedical field, using public funding. In the US for instance, each review board is responsible for a subtype of research based on content or methodology (for example there are IRBs dedicated to validating clinical trial protocols, assessing cancer treatments, examining pediatric research, and reviewing qualitative research). This traditional ethics review structure cannot accommodate big data research [2]. Big data projects often reach beyond a single institution, cut across disciplines, involve data collected from a variety of sources, re-use data not originally collected for research purposes, combine diverse methodologies, orient towards population-level research, rely on large data aggregates, and emerge from collaboration with the private sector. Given this scenario, big data projects may likely fall beyond the purview of ERCs.

Another case in which big data research does not fall under ERC purview is when it relies on anonymized data. If researchers use data that cannot be traced back to subjects (anonymized or non-personal data), then according to both the US Common Rule and HIPAA regulations, the project is considered safe enough to be granted an ethics review waiver. If instead researchers use pseudonymized (or de-identified) data, they must apply for research ethics review, as in principle the key that links the de-identified data with subjects could be revealed or hacked, causing harm to subjects. In the European Union, it would be left to each Member State (and national laws or policies at local institutions) to define whether research using anonymized data should seek ethical review. This case shows once more that current research ethics regulation is relatively loose and disjointed across jurisdictions, and may leave areas where big data research is unregulated. In particular, the special treatment given anonymized data comes from an emphasis on risk at the individual level. So far in the big data discourse, the concept of harm has been mainly linked to vulnerability in data protection. Therefore if privacy laws are respected, and protection is built into the data system, researchers can prevent harmful outcomes [40]. However, this view is myopic as it does not include other misuses of data aggregates, such as group discrimination and dignitary harm. These types of harm are already emerging in the big data ecosystem, where anonymized data reveal health patterns of a certain sub-group, or computational technologies include strong racial biases [67, 68]. Furthermore, studies using anonymized data should not be deemed oversight-free by default, as it is increasingly hard to anonymize data. Technological advancements might soon make it possible to re-identify individuals from aggregate data sets [69].

The risks associated with big data projects also increase due to the variety of actors involved in research alongside university researchers (e.g., private companies, citizen science associations, bio-citizen groups, community workers cooperatives, foundations, and non-profit organizations) [70, 71]. The novel aspect of health-related big data research compared with traditional research is that anyone who can access large amounts of data about individuals and build predictive models based on that data, can now determine and infer the health status of a person without directly engaging with that person in a research program [72]. Facebook, for example, is carrying out a suicide prediction and prevention project, which relies exclusively on the information that users post on the social network [18]. Because this type of research is now possible, and the available ethics review model exempts many big data projects from ERC appraisal, gaps in oversight are growing [17, 73]. Just as corporations can re-use publicly available datasets (such as social media data) to determine life insurance premiums [74], citizen science projects can be conducted without seeking research oversight [75]. Indeed, participant-led big data research (despite being increasingly common) is another area where the traditional overview model is not effective [76]. In addition, ERCs might consider research conducted outside academia or publicly funded institutions to be not serious. Thus ERCs may disregard review requests from actors outside the academic environment (e.g., by the citizen science or health tech start up) [77].

### Novel weaknesses: functional weaknesses

Functional weaknesses are those related to the skills, composition, and operational activities of ERCs in relation to big data research.

From this functional perspective, we argue that the ex-ante review model might not be appropriate for big data research. Project assessment at the project design phase or at the data collection level is insufficient to address emerging challenges that characterize big data projects – especially as data, over time, could become useful for other purposes, and therefore be re-used or shared [53]. Limitations of the ex-ante review model have already become apparent in the field of genetic research [78]. In this context, biobanks must often undergo a second ethics assessment to authorize the specific research use on exome sequencing of their primary data samples [79]. Similarly, in a case in which an ERC approved the original collection of sensitive personal data, a data access committee would ensure that the secondary uses are in line with original consent and ethics approval. However, if researchers collect data from publicly accessible platforms, they can potentially use and re-use data for research lawfully, without seeking data subject consent or ERC review. This is often the case in social media research. Social media data, which are collected by researchers or private companies using a form of broad consent, can be re-used by researchers to conduct additional analysis without ERC approval. It is not only the re-use of data that poses unforeseeable risks. The ex-ante approach might not be suitable to assess other stages of the data lifecycle [80], such as deployment machine learning algorithms.

Rather than re-using data, some big data studies build models on existing data (using data mining and machine learning methods), creating new data, which is then used to further feed the algorithms [81]. Sometimes it is not possible to anticipate which analytic models or tools (e.g., artificial intelligence) will be leveraged in the research. And even then, the nature of computational technologies which extract meaning from big data make it difficult to anticipate all the correlations that will emerge from the analysis [37]. This is an additional reason that big data research often has a tentative approach to a research question, instead of growing from a specific research hypothesis [82].The difficulty of clearly framing the big data research itself makes it even harder for ERCs to anticipate unforeseeable risks and potential societal consequences. Given the existing regulations and the intrinsic exploratory nature of big data projects, the mandate of ERCs does not appear well placed to guarantee research oversight. It seems even less so if we consider problems that might arise after the publication of big data studies, such as repurposing or dual-use issues [83].

ERCs also face the challenge of assessing the value of informed consent for big data projects. To re-obtain consent from research subjects is impractical, particularly when using consumer generated data (e.g., social media data) for research purposes. In these cases, researchers often rely on broad consent and consent waivers. This leaves the data subjects unaware of their participation in specific studies, and therefore makes them incapable of engaging with the research progress. Therefore, the data subjects and the communities they represent become vulnerable towards potential negative research outcomes. The tool of consent has limitations in big data research—it cannot disclose all possible future uses of data, in part because these uses may be unknown at the time of data generation. Moreover, researchers can access existing datasets multiple times and reuse the same data with alternative purposes [84]. What should be the ERCs’ strategy, given the current model of informed consent leaves an ethical gap in big data projects? ERCs may be tempted to focus on the consent challenge, neglecting other pressing big data issues [53]. However, the literature reports an increasing number of authors who are against the idea of a new consent form for big data studies [5].

A final widely discussed concern is the ERC’s inadequate expertise in the area of big data research [85, 86]. In the past, there have been questions about the technical and statistical expertise of ERC members. For example, ERCs have attempted to conform social science research to the clinical trial model, using the same knowledge and approach to review both types of research [87]. However, big data research poses further challenges to ERCs’ expertise. First, the distinct methodology of big data studies (based on data aggregation and mining) requires a specialized technical expertise (e.g., information systems, self-learning algorithms, and anonymization protocols). Indeed, big data projects have a strong technical component, due to data volume and sources, which brings specific challenges (e.g., collecting data outside traditional protocols on social media) [88, 89]. Second, ERCs may be unfamiliar with new actors involved in big data research, such as citizen science actors or private corporations. Because of this lack of relevant expertise, ERCs may require unjustified amendments to research studies, or even reject big data projects *tout-court* [36]. Finally, ERCs may lose credibility as an oversight body capable of assessing ethical violations and research misconduct. In the past, ERCs solved this challenge by consulting independent experts in a relevant field when reviewing a protocol in that domain. However, this solution is not always practical as it depends upon the availability of an expert. Furthermore, experts may be researchers working and publishing in the field themselves. This scenario would be problematic because researchers would have to define the rules experts must abide by, compromising the concept of independent review [19]. Nonetheless, this problem does not disqualify the idea of expertise but requires high transparency standards regarding rule development and compliance. Other options include *ad-hoc* expert committees or provision of relevant training for existing committee members [47, 90, 91]. Given these options, which one is best to address ERCs’ lack of expertise in big data research?

### Reforming the ERC

**Fig. 2:**
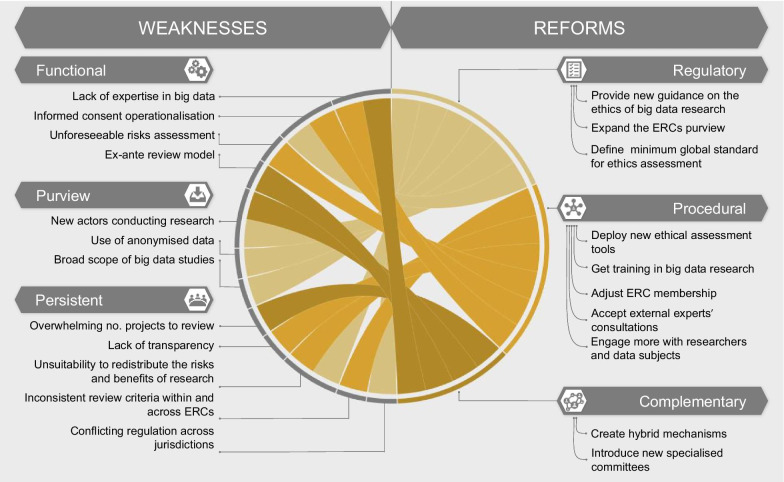
Reforms overview for the research oversight mechanism

Our analysis shows that ERCs play a critical role in ensuring ethical oversight and risk–benefit evaluation [92], assessing the scientific validity of a project in its early stages, and offering an independent, critical, and interdisciplinary approach to the review. These strengths demonstrate why the ERC is an oversight model worth holding on to. Nevertheless, ERCs carry persistent big data-specific weaknesses, reducing their effectiveness and appropriateness as oversight bodies for data-driven research. To answer our initial research question, we propose that the current oversight mechanism is not as fit for purpose to assess the ethics of big data research as it could be in principle. ERCs should be improved at several levels to be able to adequately address and overcome these challenges. Changes could be introduced at the level of the regulatory framework as well as procedures. Additionally, reforming the ERC model might mean introducing complementary forms of oversight. In this section we explore these possibilities. Figure 2 offers an overview of the reforms that could aid ERCs in improving their process.

### Regulatory reforms

The regulatory design of research oversight is the first aspect which needs reform. ERCs could benefit from new guidance (e.g., in the form of a flowchart) on the ethics of big data research. This guidance could build upon a deep rethinking of the importance of data for the functioning of societies, the way we use data in society, and our justifications for this use. In the UK, for instance, individuals can generally opt out of having their data (e.g., hospital visit data, health records, prescription drugs) stored by physicians’ offices or by NHS digital services. However, exceptions to this opt-out policy apply when uses of the data are vital to the functioning of society (for example, in the case of official national statistics or overriding public interest, such as the COVID-19 pandemic) [93].

We imagine this new guidance also re-defining the scope of ERC review, from protection of individual interest to a broader research impact assessment. In other words, it will allow the ERC’s scope to expand and to address purview issues which were previously discussed. For example, less research will be oversight-free because more factors would trigger ERC purview in the first place. The new governance would impose ERC review for research involving anonymized data, or big data research within public–private partnerships. Furthermore, ERC purview could be extended beyond the initial phase of the study to other points in the data lifecycle [94]. A possible option is to assess a study after its conclusion (as is the case in the pharmaceutical industry): ERCs could then decide if research findings and results should be released and further used by the scientific community. This new ethical guidance would serve ERCs not only in deciding whether a project requires review, but also in learning from past examples and best practices how to best proceed in the assessment. Hence, this guidance could come in handy to increase transparency surrounding assessment criteria used across ERCs. Transparency could be achieved by defining a minimum global standard for ethics assessment that allows international collaboration based on open data and a homogenous evaluation model. Acceptance of a global standard would also mean that the same oversight procedures will apply to research projects with similar risks and research paths, regardless of whether they are carried on by public or private entities. Increased clarification and transparency might also streamline the review process within and across committees, rendering the entire system more efficient.

### Procedural reforms

Procedural reforms might target specific aspects of the ERC model to make it more suitable for the review of big data research. To begin with, ERCs should develop new operational tools to mitigate emerging big data challenges. For example, the AI Now algorithmic impact assessment tool, which appraises the ethics of automated decision systems, and informs decisions about whether or not to deploy the systems in society, could be used [95]. Forms of broad consent [96] and dynamic consent [20] can also address some of the issues raised, by using, re-using, and sharing big data (publicly available or not). Nonetheless, informed consent should not be considered a panacea for all ethical issues in big data research—especially in the case of publicly available social media data [97]. If the ethical implications of big data studies affect the society and its vulnerable sub-groups, individual consent cannot be relied upon as an effective safeguard. For this reason, ERCs should move towards a more democratic process of review. Possible strategies include engaging research subjects and communities in the decision-making process or promoting a co-governance system. The recent Montreal Declaration for Responsible AI is an example of an ethical oversight process developed out of public involvement [98]. Furthermore, this inclusive approach could increase the trustworthiness of the ethics review mechanism itself [99]. In practice, the more that ERCs involve potential data subjects in a transparent conversation about the risks of big data research, the more socially accountable the oversight mechanism will become.

ERCs must also address their lack of big data and general computing expertise. There are several potential ways to bridge this gap. First, ERCs could build capacity with formal training on big data. ERCs are willing to learn from researchers about social media data and computational methodologies used for data mining and analysis [85]. Second, ERCs could adjust membership to include specific experts from needed fields (e.g., computer scientists, biotechnologists, bioinformaticians, data protection experts). Third, ERCs could engage with external experts for specific consultations. Despite some resistance to accepting help, recent empirical research has shown that ERCs may be inclined to rely upon external experts in case of need [86].

In the data-driven research context, ERCs must embrace their role as regulatory stewards, and walk researchers through the process of ethics review [40]. ERCs should establish an open communication channel with researchers to communicate the value of research ethics while clarifying the criteria used to assess research. If ERCs and researchers agree to mutually increase transparency, they create an opportunity to learn from past mistakes and prevent future ones [100]. Universities might seek to educate researchers on ethical issues that can arise when conducting data-driven research. In general, researchers would benefit from training on identifying issues of ethics or completing ethics self-assessment forms, particularly if they are responsible for submitting projects for review [101]. As biomedical research is trending away from hospitals and clinical trials, and towards people’s homes and private corporations, researchers should strive towards greater clarity, transparency, and responsibility. Researchers should disclose both envisioned risks and benefits, as well as the anticipated impact at the individual and population level [54]. ERCs can then more effectively assess the impact of big data research and determine whether the common good is guaranteed. Furthermore, they might examine how research benefits are distributed throughout society. Localized decision making can play a role here [55]. ERCs may take into account characteristics specific to the social context, to evaluate whether or not the research respects societal values.

### Complementary reforms

An additional measure to tackle the novelty of big data research might consist in reforming the current research ethics system through regulatory and procedural tools. However, this strategy may not be sufficient: the current system might require additional support from other forms of oversight to complement its work.

One possibility is the creation of hybrid review mechanisms and norms, merging valuable aspects of the traditional ERC review model with more innovative models, which have been adopted by various partners involved in the research (e.g., corporations, participants, communities) [102]. This integrated mechanism of oversight would cover all stages of big data research and involve all relevant stakeholders [103]. Journals and the publishing industry could play a role within this hybrid ecosystem in limiting potential dual use concerns. For instance, in the research publication phase, resources could be assigned to editors so as to assess research integrity standards and promote only those projects which are ethically aligned. However, these implementations can have an impact only when there is a shared understanding of best practice within the oversight ecosystem [19].

A further option is to include specialized and distinct ethical committees alongside ERCs, whose purpose is to assess big data research and provide sectorial accreditation to researchers. In this model, ERCs would not be overwhelmed by the numbers of study proposals to review and could outsource evaluations requiring specialist knowledge in the field of big data. It is true that specialized committees (data safety monitoring boards, data access committees, and responsible research and innovation panels) already exist and support big data researchers in ensuring data protection (e.g., system security, data storage, data transfer). However, something like a “data review board” could assess research implications both for the individual and society, while reviewing a project’s technical features. Peer review could play a critical role in this model: the research community retains the expertise needed to conduct ethical research and to support each other when the path is unclear [101].

Despite their promise, these scenarios all suffer from at least one primary limitation. The former might face a backlash when attempting to bring together the priorities and ethical values of various stakeholders, within common research norms. Furthermore, while decentralized oversight approaches might bring creativity over how to tackle hard problems, they may also be very dispersive and inefficient. The latter could suffer from overlapping scope across committees, resulting in confusing procedures, and multiplying efforts while diluting liability. For example, research oversight committees have multiplied within the United States, leading to redundancy and disharmony across committees [47]. Moreover, specialized big data ethics committees working in parallel with current ERCs could lead to questions over the role of the traditional ERC, when an increasing number of studies will be big data studies.

## Conclusions

ERCs face several challenges in the context of big data research. In this article, we sought to bring clarity regarding those which might affect the ERC’s practice, distinguishing between novel and persistent weaknesses which are compounded by big data research. While these flaws are profound and inherent in the current sociotechnical transformation, we argue that the current oversight model is still partially capable of guaranteeing the ethical assessment of research. However, we also advance the notion that introducing reform at several levels of the oversight mechanism could benefit and improve the ERC system itself. Among these reforms, we identify the urgency for new ethical guidelines and new ethical assessment tools to safeguard society from novel risks brought by big data research. Moreover, we recommend that ERCs adapt their membership to include necessary expertise for addressing the research needs of the future. Additionally, ERCs should accept external experts’ consultations and consider training in big data technical features as well as big data ethics. A further reform concerns the need for transparent engagement among stakeholders. Therefore, we recommend that ERCs involve both researchers and data subjects in the assessment of big data research. Finally, we acknowledge the existing space for a coordinated and complementary support action from other forms of oversight. However, the actors involved must share a common understanding of best practice and assessment criteria in order to efficiently complement the existing oversight mechanism. We believe that these adaptive suggestions could render the ERC mechanism sufficiently agile and well-equipped to overcome data-intensive research challenges and benefit research at large.

## Data Availability

Not applicable.

## Abbreviations

ERC(s): Ethics Review Committee(s)
HIPAA: Health Insurance Portability and Accountability Act
IRB(s): Institutional Review Board(s)
NHS: National Health Service
REC(s): Research Ethics Committee(s)
UK: United Kingdom
US: United States

## Footnotes

1 There is an unsettled discussion about whether ERCs ought to play a role in evaluating both scientific and ethical aspects of research, or whether these can even come apart—but we will not go into detail here. 25.Dawson AJ, Yentis SM. Contesting the science/ethics distinction in the review of clinical research. Journal of Medical Ethics. 2007;33(3):165–7, 26.Angell EL, Bryman A, Ashcroft RE, Dixon-Woods M. An analysis of decision letters by research ethics committees: the ethics/scientific quality boundary examined. BMJ Quality & Safety. 2008;17(2):131–6.
